# Le vaccin antipaludique RTS,S/AS01 chez les enfants âgés de 5 à 17 mois au moment de la première vaccination

**DOI:** 10.11604/pamj.2018.30.142.13152

**Published:** 2018-06-19

**Authors:** Pascale Vandoolaeghe, Lode Schuerman

**Affiliations:** 1 GSK Vaccines, Wavre, Belgium

**Keywords:** Paludisme, Plasmodium falciparum, RTS, S/AS01, efficacité, innocuité, Malaria, plasmodium falciparum, RTS, S/AS01, effectiveness, safety

## Abstract

Le vaccin antipaludique RTS,S/AS01 a reçu un avis scientifique favorable de l’Agence Européenne des Médicaments (EMA) en Juillet 2015. L’Organisation Mondiale de la Santé (OMS) a recommandé l’introduction pilote de ce vaccin chez des enfants âgés d’au moins 5 mois en utilisant un schéma de vaccination comprenant 3 doses initiales espacées d’au moins un mois et une 4^ème^ dose administrée 15 à 18 mois après la 3^ème^ dose. Des essais cliniques et des modèles mathématiques ont montré que la protection partielle contre le paludisme conférée par le vaccin RTS,S/AS01 pourrait avoir un impact substantiel sur la santé publique si le vaccin est utilisé en association avec d’autres mesures de lutte antipaludique, en particulier dans les zones hautement endémiques. L’impact le plus important a été observé chez les enfants âgés de 5 mois ou plus ayant reçu 4 doses de RTS,S/AS01. Le vaccin sera ensuite évalué en situation réelle afin de déterminer son impact sur la mortalité, son innocuité dans le cadre d’une vaccination de routine, et la faisabilité opérationnelle d’administrer 4 doses du vaccin dont certaines nécessitant de nouveaux contacts dans le calendrier de vaccination. En cas de succès, cela permettra une mise en œuvre à plus grande échelle.

## Abstract (en)

RTS,S/AS01 malaria vaccine received a favorable scientific opinion from the European Medicines Agency (EMA) in July 2015. The World Health Organization (WHO) recommended the pilot introduction of this vaccine in children aged 5 months or older using a vaccination schedule which included 3 initial doses separated by at least 1 month and a 4^th^ dose administered 15-18 months after the 3^rd^ dose. Clinical trials and mathematical models showed that the partial protection against malaria conferred by vaccine RTS,S/AS01 Malaria Vaccine could have a substantial impact on public health if the vaccine was used in association with other control measures for malaria, in particular in highly endemic areas. The most significant impact was observed in children aged 5 months or older who received 4 doses of RTS,S/AS01. Vaccine effectiveness will then be evaluated under real-life conditions in order to determine its impact on mortality, its safety in the context of routine immunization and the feasibility of implementing the 4-dose vaccination schedule requiring new immunization contacts. If successful, this would pave the way for larger-scale implementation.

## Introduction

Le paludisme est l’un des plus grands défis sanitaires de l’histoire de l’humanité. Selon les données épidémiologiques de l’Organisation Mondiale de la Santé (OMS), le paludisme a causé plus de 430 000 décès en 2015 [1], malgré les progrès significatifs en termes de lutte antipaludique au cours des dernières décennies. Chez l’homme, la maladie est causée par 5 espèces du genre *Plasmodium*, dont *Plasmodium falciparum* causant seul plus de 90% des décès reliés au paludisme. Suite à la mise en œuvre de mesures préventives recommandées par l'OMS, l'incidence du paludisme et les taux de mortalité ont considérablement diminué au cours des 15 dernières années. Cependant, la transmission, la morbidité et la mortalité du paludisme restent élevées dans de nombreuses zones endémiques [1]. Malgré la diminution de la prévalence de la parasitémie de *P. falciparum* chez les enfants âgés de 2 à 10 ans entre 2000 et 2010, 57% de la population africaine vit toujours dans des zones de transmission hyper- ou holo-endémique [2]. Le paludisme est actuellement la 4^ème^cause de mortalité chez les enfants de moins de 5 ans en Afrique subsaharienne [1]. Les mesures actuelles de lutte antipaludique reposent en grande partie sur la chimioprévention, incluant un traitement préventif intermittent durant la grossesse et chez les nourrissons, et la chimioprévention saisonnière du paludisme chez les enfants. La chimioprévention consiste à administrer un traitement complet de sulfadoxine-pyrimenthamine et parfois d’amodiaquine aux populations ciblées, quelle que soit leur parasitémie. Cependant, le parasite Plasmodium est notoire pour sa capacité à développer une résistance aux traitements. Une résistance à l’artémisinine, qui est l’un des composants principaux des traitements antipaludiques recommandés en première intention, a été reportée dans 5 pays d’Asie du Sud-Est. La propagation de la résistance à l’artémisinine en Afrique pourrait avoir des conséquences graves pour la santé publique.

Des mesures de lutte antivectorielle ont également été mises en œuvre, telles que l’utilisation de moustiquaires imprégnées d'insecticide (MIIs) ou la pulvérisation intradomiciliaire (PID) d’insecticides à effet rémanent. Bien que ces mesures aient permis de réduire considérablement la mortalité et la morbidité en Afrique au cours des 10 dernières années [3,4], des taux croissants de résistance aux insecticides ont été rapportés chez les moustiques vecteurs responsables de la transmission du paludisme [5]. Cette menace a été reconnue par l'OMS qui a publié un plan mondial pour la gestion de la résistance aux insecticides en Mai 2012. Jusqu’il y a peu, le développement d'un vaccin efficace restait un objectif non atteint dans la lutte contre le paludisme. Le seul vaccin candidat ayant achevé l’évaluation de phase III est le vaccin RTS,S/AS01 (*Mosquirix*, GSK Vaccines). Bien que son utilisation ne soit pas encore autorisée par les autorités réglementaires nationales en Afrique subsaharienne, le vaccin RTS,S/AS01 a reçu un avis scientifique favorable de l'Agence Européenne des Médicaments (EMA) en juillet 2015 pour la vaccination des enfants âgés de 6 semaines à 17 mois [6]. Ensuite, l’OMS a recommandé l’introduction du vaccin chez les enfants âgés de 5 à 17 mois dans le cadre d’une série de projets pilotes utilisant un schéma d’administration à 4 doses (3 doses initiales espacées d’au moins 4 semaines, administrées de préférence avant l’âge de 9 mois, suivies d’une 4^ème^ dose administrée 15 à 18 mois après la 3^ème^ dose) dans les régions où la transmission du paludisme est modérée à élever [7]. Cet article passe en revue les données disponibles sur RTS,S/AS01 dans la population ciblée par les projets pilotes.

## Méthodes

### Description du vaccin

Le vaccin antipaludique RTS,S/AS01 cible le stade pré-érythrocytaire du parasite [8]. Une description détaillée de la formulation finale et des formulations antérieures du vaccin, ainsi qu’une analyse des études réalisées chez les adultes et des essais de phase I et II chez les enfants, ont été publiées précédemment [9]. En bref, le vaccin se compose d’une protéine recombinante formant spontanément des pseudo-particules virales, appelée RTS,S, associée au système adjuvant breveté AS01E. L'antigène RTS,S contient une portion de la protéine circumsporozoite de *P. falciparum* (CS) fusionnée à l'extrémité amino-terminale de l'antigène de surface du virus de l'hépatite B (HBsAg), qui est également utilisé dans les vaccins homologués contre l'hépatite B. Pour stabiliser les particules recombinantes, la protéine de fusion est co-exprimée avec la protéine HBsAg (S) dans *Saccharomyces cerevisiae*. L’AS01E est constitué de 3-O-désacyl-4’-monophosphoryl-lipide-A (MPL; produit par GSK), de QS-21 (Quillaja saponaria Molina, fraction 21; sous licence par GSK d’Antigenics Inc, une filiale en propriété exclusive d’Agenus Inc., une société de Delaware, États-Unis) et de liposomes. Le vaccin RTS,S/AS01 consiste en une poudre (RTS,S lyophilisé) et une suspension (AS01E) présentées dans 2 flacons séparés sans agent conservateur. Le vaccin final est obtenu en reconstituant la poudre avec la suspension pour donner 1mL de liquide opalescent, incolore à brun pâle. Une dose de vaccin est obtenue en prélevant 0,5mL du flacon après reconstitution. Chaque dose contient 25 μg d’antigène RTS,S, ainsi que 25 μg de chaque molécule immunomodulatoire MPL et QS21. Le vaccin est administré par voie intramusculaire [10].

### Divulgation des intérêts financiers et concurrents

GlaxoSmithKline Biologicals SA a payé tous les coûts associés au développement et à la publication du présent manuscrit. PV et LS sont des employés du groupe de sociétés GSK. PV et LS possèdent des options d'achat d'actions / des actions restreintes dans le groupe de sociétés GSK.

### Etat actuel des connaissances

### Essais cliniques

Parmi plus de 30 vaccins candidats antipaludiques [11], RTS,S/AS01 est le seul ayant atteint un stade d’évaluation clinique avancé, réalisée conformément aux directives de l'OMS garantissant la qualité, l’innocuité et l’efficacité des vaccins recombinants ciblant les parasites Plasmodium aux stades pré-érythrocytaire et sanguin [12]. Une revue systématique du développement clinique du vaccin antipaludique RTS,S/AS fourni une vue d’ensemble récente des résultats de toutes les études de phases II et III [13], indépendamment de la formulation du vaccin ou de la population ciblée. Dans cet article, nous nous concentrons sur les résultats obtenus chez les enfants âgés de 5 à 17 mois au moment de la première vaccination, la tranche d'âge pour laquelle l’implémentation pilote a été recommandée par l'OMS. Un résumé des essais de phase II et III dans cette catégorie d’âge est présenté dans le Tableau 1 [14-34]. L’efficacité vaccinale (EV) et d’autres objectifs ont été évalués dans l’étude Malaria-055 (ClinicalTrials.gov: NCT00866619) [24]. Cet essai de phase III a été mené dans 7 pays d’Afrique subsaharienne chez 6 537 nourrissons âgés de 6 à 12 semaines et 8 922 enfants âgés de 5 à 17 mois au moment de la première vaccination (Tableau 1) [14-34]. Les participants ont été répartis aléatoirement (1:1:1) afin de recevoir 4 doses de RTS,S/AS01, 3 doses de RTS,S/AS01 et 1 dose de vaccin témoin, ou 4 doses de vaccins témoin aux mois 0, 1, 2 et 20 de l’étude [24]. Chez les enfants vaccinés à l’âge de 5 à 17 mois, le vaccin témoin était le vaccin antirabique pour les 3 premières doses et le vaccin conjugué contre les méningocoques de sérogroupe C (MenC) pour la 4^ème^ dose [24], et la durée de suivi moyenne après la première vaccination a été de 48 mois [25]. Tous les participants ont eu accès à des MIIs et leur utilisation a été évaluée pendant toute la durée de l’étude. Les taux d’utilisation des MIIs ont été élevés et similaires dans les différents groupes. 14 mois après la première dose, plus de 78% des enfants des groupes RTS,S/AS01 et témoin utilisaient des MIIs et les taux de couverture sont restés élevés (environ 74%) jusqu’à la fin de l’étude [**25**].

**Tableau 1:** Résumé des essais cliniques de phase II et III pour le vaccin RTS,S/AS01 chez les enfants de 5 à 17 mois au moment de la première dose

Objectifs	Conception de l'étude et population	Groupes (N)	Refs.
Malaria-047 (NCT00360230)			
Innocuité de 2 formulations vaccinales administrées selon plusieurs calendriers vaccinaux Innocuité et immunogénicité	Étude de Phase II, partiellement en aveugle (aveugle pour la formulation du vaccin, ouvert au calendrier vaccinal), randomisée en 6 groupes, contrôlée, multicentrique. Enfants, sains, âgés de 5 à 17 mois, du Ghana. Calendrier vaccinal : 0–1 mois, 0–1–2 mois, 0–1–7 mois	RTS,S/AS01, 0–1 (90); RTS,S/AS02, 0–1 (90); RTS,S/AS01, 0–1–2 (90); Vaccin antirabique, 0–1–2 (45); RTS,S/AS02, 0–1–2 (45); RTS,S/AS01, 0–1–7 (90); RTS,S/AS02, 0–1–7 (90)	[14,15]
Malaria-049 (NCT00380393)			
Efficacité contre le paludisme clinique Innocuité et immunogénicité	Étude de Phase IIb, en double-aveugle, randomisée en 2 groupes, contrôlée, multicentrique, multinationale. Enfants, sains, âgés de 5 à 17 mois, de Tanzanie et du Kenya. Calendrier vaccinal : 0–1–2 mois	RTS,S/AS01 (447); Vaccin antirabique (447)	[16,17,18, 19,20,21, 22,23]
Malaria-055 (NCT00866619)			
Efficacité contre le paludisme clinique Efficacité contre le paludisme grave Rôle de la 4^ème^ dose Efficacité contre les hospitalisations et la mortalité	Étude de Phase III, en double-aveugle, randomisée en 3 groupes, contrôlée, multicentrique, multinationale. Enfants, sains, âgés de 5 à 17 mois et nourrissons de 6 à 12 semaines du Burkina Faso, Gabon, Ghana, Kenya, Malawi, Mozambique, Tanzanie. Calendrier vaccinal : 0–1–2–20 mois	Enfants âgés de 5 à 17 mois : 3 doses de RTS,S/AS01* (2976) ; 4 doses de RTS,S/AS01* (2972) ; Vaccin antirabique (Témoin)* (2974). Nourrissons âgés de 6 à 12 semaines : 3 doses de RTS,S/AS01* (2180) ; 4 doses de RTS,S/AS01* (2178) ; MenC (Témoin)* (2179)	[24-26, 27,28,29, 30-32]
Malaria-058 (NCT01148459)			
Innocuité et immunogénicité chez des nourrissons et des enfants infectés par le VIH	Étude de Phase III, en double-aveugle, randomisée en 2 groupes, contrôlée, multicentrique. Nourrissons et enfants de sexe masculin et féminin âgés de 6 semaines à 17 mois infectés par le VIH, du Kenya. Calendrier vaccinal: 0–1–2 mois	RTS,S/AS01 (99); Vaccin antirabique (101)	[33]
Malaria-061 (NCT01323972)			
Consistance des lots et non infériorité de la réponse immunitaire anti-CS induite par le RTS,S/AS01 (3 lots de fabrication à l’échelle commerciale) comparé à un lot de RTS,S/AS01 fabriqué à l’échelle pilote Innocuité et immunogénicité	Étude de Phase III, en double-aveugle, randomisée en 4 groupes, multicentrique. Enfants de sexe masculin et féminin, sains, âgés de 5 à 17 mois, du Nigeria Calendrier vaccinal: 0–1–2 mois	Lots à l’échelle commerciale : RTS,S/AS01, lot 1 (81) ; RTS,S/AS01, lot 2 (79); RTS,S/AS01, lot 3 (80) Lot à l’échelle de Phase III : RTS,S/AS01 témoins (80)	[34]

* groupe à 4 doses : enfants recevant 4 doses de RTS,S/AS01 aux mois 0, 1, 2 et 20 ; groupe à 3 doses : enfants recevant 3 doses de RTS,S/AS01 aux mois 0, 1 et 2 et le vaccin témoin au mois 20 ; groupe témoin : enfants recevant 4 doses de vaccin témoin aux mois 0, 1, 2 et 20. Le vaccin témoin au mois 20 est MenC pour les 2 catégories d’âge.

CS : protéine circumsporozoite ; MenC, vaccin conjugué contre les méningocoques de sérogroupe C ; N, nombre de participants dans chaque groupe.

### Efficacité clinique

#### EV contre le paludisme clinique

**Figure 1:**
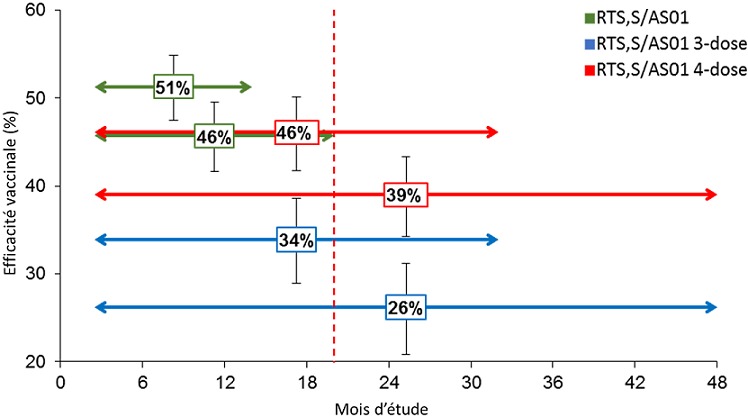
Efficacité vaccinale contre tous les épisodes de paludisme clinique pendant différentes périodes de suivi depuis la 3ème dose dans l’essai de phase III, pour les enfants âgés de 5 à 17 mois (cohorte conforme au protocole)

**Figure 2:**
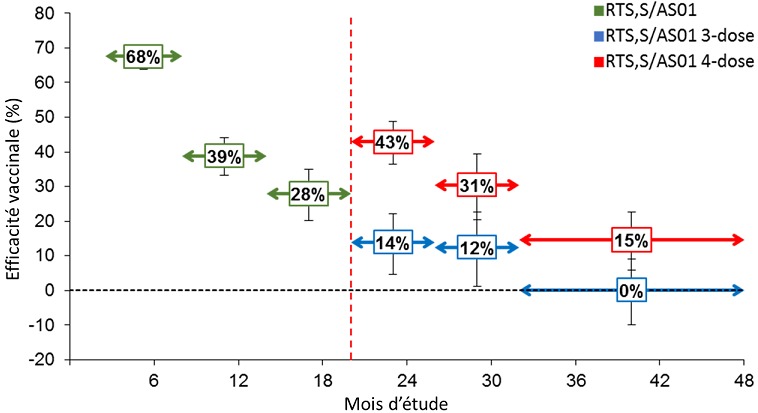
Efficacité vaccinale contre tous les épisodes de paludisme clinique, exprimée en incidence comparative du paludisme clinique stratifié pour des périodes de suivi de 6 mois dans l’essai de phase III, pour les enfants âgés de 5 à 17 mois (cohorte conforme au protocole)

**Figure 3:**
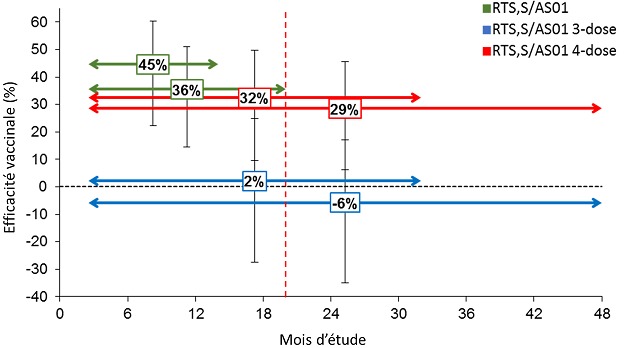
Efficacité vaccinale contre les épisodes de paludisme grave pendant différentes périodes de suivi depuis la 3ème dose dans l’essai de phase III, pour les enfants âgés de 5 à 17 mois (cohorte conforme au protocole)

**Figure 4:**
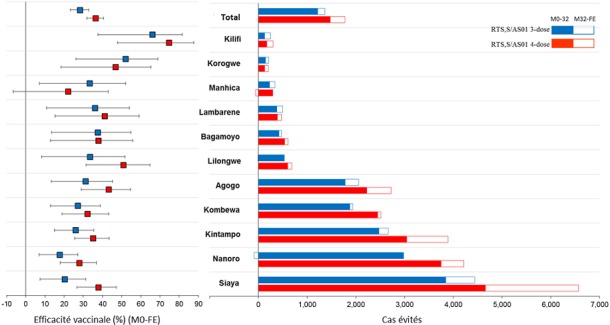
Efficacité vaccinale au cours d’une période de suivi moyenne de 48 mois et cas de paludisme clinique évités pour 1000 enfants vaccinés au cours de l’essai de phase III, par centre, pour les enfants âgés de 5 à 17 mois (cohorte en intention-de-traitement)

Les objectifs de l'étude d'efficacité de phase III comprenaient l'évaluation de l’EV contre différentes présentations cliniques du paludisme (voir Annexe 1 pour les définitions principales des cas cliniques) sur différents intervalles de temps [24]. L’EV contre tous les cas de paludisme a été évaluée par le ratio du taux d’incidence (groupe vaccin/ groupe témoin), en utilisant un modèle de régression binomiale négative pour contrôler l'interdépendance entre les cas observés chez un même participant [26]. Les analyses d’efficacité ont été effectuées sur la cohorte conforme au protocole (CAP), comprenant les enfants ayant reçu au moins 3 doses de RTS,S/AS01 conformément au protocole et ayant contribué à la surveillance de l'efficacité à partir du 14^ème^jour après la dose 3, et sur la cohorte en intention-de-traitement (IDT) [35], comprenant les enfants ayant reçu au moins 1 dose de RTS,S/AS01. Les estimations d’EV contre le paludisme clinique sur différents intervalles de temps étaient similaires pour les analyses réalisées sur les cohortes CAP (Figure 1) et IDT [35]. Dans les Figure 1, Figure 2 et Figure 3, « RTS,S/AS01 » comprend les groupes RTS,S/AS01 3-dose et 4-dose avant l’administration de la 4^ème^ dose; « RTS,S/AS01 3-dose » correspond au groupe des enfants ayant reçu 3 doses de RTS,S/AS01 aux mois 0, 1 et 2 et le vaccin témoin au mois 20 ; et « RTS,S/AS01 4-dose » correspond au groupe des enfants ayant reçu les 4 doses de RTS,S/AS01 aux mois 0, 1, 2 et 20. Les flèches doubles indiquent la période de suivi au cours de laquelle les estimations d'efficacité du vaccin respectives ont été calculées. Les barres d'erreur indiquent les intervalles de confiance à 95%. La ligne verticale pointillée indique le moment de l’administration de la 4^ème^ dose. Jusqu'au 20ème mois, les résultats des 2 groupes RTS,S/AS01 ont été regroupés car tous les enfants avaient reçu 3 doses de vaccin. Un schéma de vaccination à 3 doses a permis d'obtenir une EV importante contre le paludisme clinique pendant toute la durée de l'étude. La protection était la plus élevée immédiatement après la vaccination, avec 51,3% d'efficacité (intervalle de confiance [IC] à 95%: 47,5 – 54,9) au cours des 12 premiers mois de suivi, et a diminué avec le temps. Après 18 mois de suivi, avant l’administration de la 4^ème^ dose, l’EV était de 45,7% (IC à 95%: 41,7 – 49,5). Pour toute la période de l’étude, après une durée de suivi moyenne de 48 mois, l’EV était de 26,2% (IC à 95%: 20,8 – 31,2) chez les enfants n’ayant reçu que 3 doses de vaccin et de 39,0% (IC à 95%: 34,3 – 43,3) chez les enfants ayant reçu la 4ème dose [6] (Figure 1). L'incidence de la maladie a également été comparée entre les groupes vaccinés et témoins pour des périodes successives de 6 mois [36] (Figure 2). La légende de la Figure 2 est identique à la légende de la Figure 1, expliquée précédemment. Cette analyse *post-hoc* doit être interprétée avec prudence puisque les groupes n'ont été randomisés qu’au début de l'étude, avant la vaccination, et pourraient ne plus être entièrement comparables après certains intervalles en raison de l’exposition différentielle à P. falciparum. Cette analyse comparative fournit néanmoins un aperçu supplémentaire de l'évolution potentielle de l'EV au fil du temps. L'EV contre le paludisme clinique était de 67,6% (IC à 95% : 63,8 – 71,0) au cours des 6 premiers mois et a diminué progressivement pendant les intervalles ultérieurs pour atteindre 0,1% (IC à 95% : -9,9–9,1) au cours de la dernière période considérée pour les enfants n’ayant pas reçu la 4^ème^ dose (à partir de 30 mois après la 3^ème^ dose jusqu'à la fin de l'essai). Pendant la période de 6 mois suivant la 4^ème^ dose de RTS,S/AS01, l’EV contre le paludisme clinique était de 42,9% (IC à 95% : 36,4 – 48,7) et a baissé jusqu’à 14,6 % (IC à 95%: 5,8 – 22,6) pendant le dernier intervalle de l’étude (Figure 2). Ces résultats renforcent la conclusion qu'une 4^ème^ dose est nécessaire pour maintenir une EV plus élevée et de plus longue durée. Ces estimations de l’EV étaient cohérentes avec celles d'un essai de phase II chez des enfants de 5 à 17 mois, dans lequel l'efficacité de 3 doses de RTS,S/AS01 a été évaluée pendant 7 ans. Après environ 12 mois de suivi, l’EV contre les cas de paludisme clinique (parasitémie de *P. falciparum* =2 500/mm^3^ et température = 37,5°C) était de 42% (IC à 95% : 22 – 57) [16]. L’EV a baissé jusqu’à 23,5% (IC à 95% : -0,7–41,9) pendant les 4 premières années de suivi [37] et jusqu’à 7,0% (IC à 95%:-14,5 – 24,6) pendant la durée de suivi totale de 7 ans [38]. L’EV chez les nourrissons âgés de 6 à 12 semaines a également été évaluée dans l'essai de phase III et s'est avérée être considérablement plus faible que chez les enfants plus âgés. Sur toute la période de l’essai (durée médiane de suivi de 38 mois après la première dose), l’EV contre tous les cas de paludisme clinique était de 18,2% (IC à 95% : 11,4–24,5) dans le groupe à 3 doses et de 26,7% (IC à 95% : 20,5–32,4) dans le groupe ayant reçu une 4ème dose [6]. Les causes de l'efficacité plus basse chez les nourrissons ne sont pas encore bien comprises, mais pourraient inclure l'immaturité relative du système immunitaire des nourrissons, un effet inhibiteur des anticorps maternels, une certaine interférence immunitaire due à l’administration simultanée des autres vaccins pédiatriques de routine, et/ou l'effet suppressif de l'exposition aux antigènes du paludisme *in utero* [27]. En conséquence, l'OMS n'a pas recommandé l'inclusion de RTS,S/AS01 dans le calendrier vaccinal du Programme Élargi de Vaccination (PEV) pour les nourrissons de 6 à 12 semaines en Afrique [7]. Des analyses univariées et multivariées ont suggéré que l'efficacité était semblable chez les enfants ayant commencé la vaccination à l'âge de 5 à 11 mois ou à l'âge de 12 à 17 mois [25]. Cette constatation a été prise en compte par l'OMS lorsqu'elle a recommandé de commencer la vaccination le plus tôt possible après l'âge de 5 mois. Dans une analyse univariée par sexe, les estimations d’EV jusqu'à 32 mois après la 3^ème^ dose étaient légèrement plus élevées chez les garçons que chez les filles dans le groupe à 3 doses (37% et 32%, respectivement) et dans le groupe à 4 doses (43% et 35%, respectivement) [7]. Des analyses multivariées ont suggéré que l’augmentation de l’EV observée après la 4^ème^ dose était significativement plus élevée chez les garçons que chez les filles. Cependant, en tenant compte de toutes les analyses effectuées par sexe, y compris celles chez les nourrissons, aucune tendance constante démontrant une différence d’efficacité entre les garçons et les filles n’apparait. Aucune évidence n'a été trouvée concernant l'impact d'autres mesures de lutte antipaludique (MIIs et PID), de l'état nutritionnel de l'enfant et de la distance de l'établissement de soins le plus proche sur l’EV contre le paludisme clinique. Dans les essais de phase II et III, aucun impact majeur sur l’EV n’a été observé en utilisant d’autres seuils de densité parasitaire pour définir les cas de paludisme, ce qui suggère que toute définition de cas avec parasitémie positive est suffisamment spécifique pour éviter une estimation biaisée de l’EV [17,18,25]. Cela peut s'expliquer par le mécanisme d'action du vaccin, qui est pré-érythrocytaire et limite l'infection en empêchant la libération de mérozoïtes du foie, mais ne modifie pas la progression de la maladie et la densité parasitaire une fois que l'infection a atteint le stade sanguin. Dans l'étude d'efficacité de phase III, l’EV a également été évaluée par centre d'étude (Figure 4). Dans la Figure 4, « RTS,S/AS01 3-dose » correspond au groupe des enfants ayant reçu 3 doses de RTS,S/AS01 aux mois 0, 1 et 2 et le vaccin témoin au mois 20, et « RTS,S/AS01 4-dose correspond au groupe des enfants ayant reçu les 4 doses de RTS,S/AS01 aux mois 0, 1, 2 et 20; « M » signifie « mois » et « FE » signifie la fin de l’étude (en moyenne 48 mois après la première vaccination). Les barres d'erreur indiquent les intervalles de confiance à 95%. Les centres d’études sont listés par incidence croissante de paludisme clinique mesurée chez les nourrissons du groupe témoin durant les 12 premiers mois de suivi. L'efficacité variait significativement selon le centre pendant les 18 mois de suivi après la 3ème dose, avec des valeurs d’EV allant de 40,2% (IC à 95% : 28,5 – 49,9) à Kombewa, Ouest du Kenya à 77,4% (IC à 95%: 26,4 – 93,1) à Kilifi, zone côtière du Kenya, mais restait statistiquement significative dans tous les centres et au travers des différentes intensités de transmission [27]. L’EV avait tendance à être plus faible dans les centres avec une incidence du paludisme plus élevée, bien que des analyses multivariées n'aient révélé aucune interaction statistiquement significative entre l’EV et la transmission [27]. Une tendance similaire avait été observée dans une étude de phase II analysant l’EV en fonction du niveau d'exposition au paludisme [37,38], et dans des prévisions de modélisation montrant une plus faible efficacité du vaccin dans les situations de transmission élevée [39].

#### EV contre le paludisme grave

L’EV contre le paludisme grave sur différentes périodes de suivi est présentée dans la Figure 3. La légende de la Figure 3 est identique à la légende de la Figure 1, expliquée précédemment. Les estimations d’EV étaient cohérentes pour les analyses CAP et IDT (35). À la fin de l'étude, l’EV contre le paludisme grave n'était plus détectable dans le groupe à 3 doses, mais persistait dans le groupe à 4 doses, ce qui confirme l'importance de la 4^ème^ dose. Bien qu'aucune corrélation significative n'ait été établie, les estimations d’EV semblaient plus élevées dans les centres ayant une intensité de transmission plus faible, ce qui est semblable aux tendances observées pour l’EV contre le paludisme clinique [25]. Néanmoins, aucune conclusion définitive n'a pu être tirée concernant l’EV contre le paludisme grave par centre d'étude, en raison du faible nombre de cas résultant en de larges intervalles de confiance. 97% des enfants hospitalisés pour un cas de paludisme grave se sont rétablis sans séquelles avant la fin de l'étude, tandis que les 3% restants sont décédés ou ont survécu avec des séquelles [25]. Le taux de létalité du paludisme grave observé dans l’étude Malaria-055 est à l'extrémité inférieure de la fourchette de 3 à 13% précédemment reportées pour des patients hospitalisés pour paludisme grave [40-42], incluant un taux de létalité de 8,5% chez les patients traités par l’artésunate intraveineux [43]. Une constatation importante est que, contrairement aux résultats jusqu'à la 4ème dose, aucune réduction significative du nombre de cas de paludisme grave n'a été observée chez les enfants du groupe à 3 doses sur toute la période d'étude. Pour les enfants n'ayant pas reçu la 4^ème^ dose, un risque plus élevé de paludisme grave est apparu à partir du 21^ème^ mois jusqu’à la fin de l'étude, en particulier dans les centres avec un taux élevé de transmission du paludisme [25]. Cela pourrait être compatible avec un effet rebond apparaissant lorsque la protection induite par le vaccin diminue après une période initiale de risque réduit d'infection par *P. falciparum*, menant à un retard dans l'acquisition de l'immunité naturelle. Cependant, le nombre plus élevé de cas graves dans ce groupe pourrait aussi être due au hasard puisque l'incidence du paludisme grave était faible et très variable dans le temps pour les 3 groupes. De plus, aucune augmentation du nombre de cas cliniques de paludisme n'a été signalée au cours de la même période de l'étude. Néanmoins, dans un essai de phase II chez des enfants de la même catégorie d'âge, une période de risque accru de paludisme clinique avait été observée au cours de la 5^ème^ année après la primovaccination [38]. Une 4^ème^ dose de RTS,S/AS01 permet d’améliorer et d’étendre la protection contre le paludisme grave tout en évitant l’augmentation du risque pendant au moins 2 ans après cette 4^ème^ dose.

#### Autres critères d’évaluation de l’EV

L’EV de RTS,S/AS01 contre les hospitalisations dues au paludisme a également été évaluée et était de 41,5% (IC à 95%: 29,1 – 51,7) après 18 mois de suivi. Au cours d'un suivi médian de 30 mois, les estimations d’EV étaient de 18,1% (IC à 95%: 1,1 – 32,3) dans le groupe à 3 doses et de 40,1% (IC à 95%: 26,2–51,5) dans le groupe à 4 doses. Sur toute la période d'étude, l’EV n'était plus statistiquement significative chez les enfants n'ayant reçu que 3 doses (12,1%, IC à 95%: -5,0 – 26,4), mais restait significative dans le groupe ayant reçu la 4^ème^ dose (37,2%, IC à 95%: 23,6 – 48,5) [6]. Dans l’étude prospective de phase III, l’EV contre la mortalité toutes causes confondues ou contre la mortalité liée au paludisme n'a pas été démontrée. En effet, un faible taux de létalité a été observé dans tous les groupes, probablement en raison de la qualité des soins de santé reçus par les enfants inclus dans cet essai. Un des centres de l’étude a rapporté une réduction de 70% de la mortalité toutes causes confondues chez les participants par rapport aux enfants qui n’étaient pas inclus dans l’étude [44]. L’absence d’impact de RTS,S/AS01 sur la mortalité dans cette étude n'exclut cependant pas un impact potentiel dans des conditions de terrain. L’implémentation pilote et les études de phase IV permettront d'étudier davantage l’EV en matière de mortalité.

#### EV contre les différentes souches de P. falciparum

Il existe différentes variantes de la protéine CS dans les populations de *P. falciparum*. Cependant, la région centrale de la protéine CS contenant des répétitions d'acides aminés NANP, incluse dans la protéine de fusion RTS, est l'épitope principal des cellules B, et est bien conservée dans les différentes souches de *P. falciparum*. La région C-terminale de la protéine CS est plus polymorphe et contient les épitopes de cellules T Th2R et Th3R, également inclus dans la protéine de fusion RTS. L’analyse des séquences Th2R et Th3R de parasites isolés chez des adultes semi-immuns ou chez des enfants de 1 à 4 ans inclus dans les essais de phase II n'a montré aucune différence significative en termes de prévalence des séquences contenues dans le vaccin ou d'autres allèles entre les individus ayant reçu le vaccin RTS,S/AS et le vaccin témoin [45-47]. Une étude plus récente a évalué le polymorphisme génétique de la protéine CS de *P. falciparum* prélevés chez des enfants infectés des groupes RTS,S/AS01 et témoin du grand essai d'efficacité de phase III [28]. Les résultats ont confirmé que RTS,S/AS01 offrait au minimum une protection partielle contre toutes les souches de *P. falciparum*, mais semblait être significativement plus efficace pour prévenir le paludisme causé par des parasites avec une séquence CS correspondante à la souche vaccinale. Par conséquent, l'efficacité globale de RTS,S/AS01 pourrait dépendre de la proportion de parasites correspondants à la souche vaccinale, qui était de moins de 10% dans cette étude [28]. Cela signifie que l’EV de RTS,S/AS01 observée dans l'essai de phase III reflète son efficacité dans un environnement avec seulement une faible proportion de parasites correspondant à la souche vaccinale.

#### Impact sur la santé publique

L'efficacité vaccinale est une mesure importante pour comprendre l'impact potentiel d'un vaccin, mais elle reste une valeur relative qui est seulement indicative de la proportion de cas de maladie évités. L'EV ne donne pas d'information sur le nombre absolu de cas qu'un vaccin peut prévenir. Il est donc également important de calculer le nombre absolu de cas de paludisme évités suite à la vaccination par RTS,S/AS01 lors de l'essai de phase III, afin de mieux comprendre ses avantages potentiels pour la santé publique. Le nombre de cas pouvant être évités par une intervention dépend directement du nombre de cas survenant dans la population (incidence de base). Dans l'essai de phase III, l'impact de RTS,S/AS01 sur le paludisme a donc été évalué par centre d'étude, et est exprimé comme le nombre de cas évités, correspondant à la somme des différences trimestrielles de l'incidence des cas entre les enfants du groupe témoin et les enfants vaccinés (cohorte IDT). Sur la durée de l'étude, le nombre de cas de paludisme clinique évités pour 1 000 enfants vaccinés variait parmi les centres d’étude entre 215 et 4 443 (1 363 cas en moyenne, IC à 95%: 995 – 1 797) dans le groupe à 3 doses et entre 205 et 6 565 (1 774 cas en moyenne, IC à 95%: 1 387 – 2 186) dans le groupe à 4 doses (Figure 4). Bien que le nombre de cas de paludisme évités ait continué à s’accumuler jusqu'à la fin de l'étude dans les 2 groupes, la 4ème dose de vaccin a entraîné une augmentation du bénéfice en termes d'impact par rapport au schéma de vaccination à 3 doses. L'impact le plus important en termes de nombre de cas de paludisme évités a été observé dans les régions où l'incidence du paludisme était le plus élevée [25]. Un impact positif du RTS,S/AS01 a été observé sur toute la période d'étude en termes de nombre de cas de paludisme grave évités par 1 000 enfants ayant reçu 4 doses de vaccin [25]. En raison de la plus faible incidence du paludisme grave, aucune conclusion ne peut être tirée de l'analyse par centre d'étude. Quatre groupes indépendants ont élaboré des modèles mathématiques pour évaluer l'impact de RTS,S/AS01 sur la santé publique et le rapport coût-efficacité [48]. Les données de l'essai de phase III ont été utilisées pour paramétrer ces modèles. Un ensemble d'hypothèses harmonisées à la suite d'une initiative de l'OMS a été utilisé, comprenant les paramètres démographiques et l'accès aux traitements antipaludiques, et tenant compte des différentes intensités de transmission du paludisme. Les 4 modèles ont tous prédit une efficacité initiale élevée, suivie par un déclin rapide au cours des 12 premiers mois [49]. Tous les modèles ont prévu la possibilité d'un déplacement de l'âge du paludisme vers des individus plus âgés, en supposant que la combinaison de toutes les mesures préventives mises en œuvre dans les programmes de lutte contre le paludisme retarderait l'acquisition de l'immunité naturelle.

### Immunogénicité

**Figure 5:**
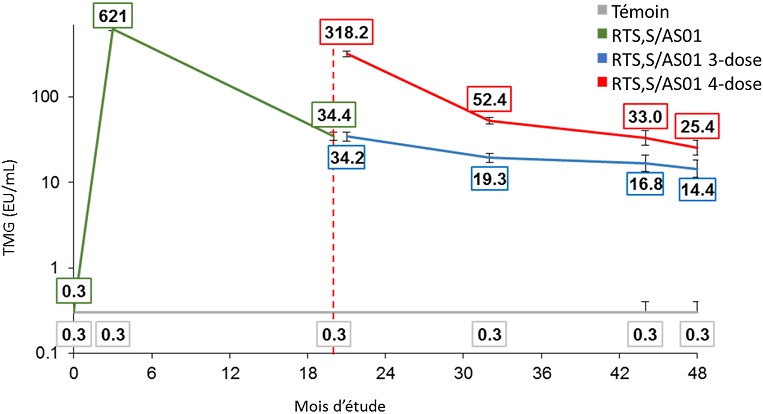
TMGs d’anticorps anti-CS pour les enfants âgés de 5 à 17 mois (cohorte conforme au protocole pour l’immunogénicité)

Dans l'essai de phase III, l'immunogénicité a été évaluée en mesurant les concentrations d'anticorps anti-CS dans la cohorte CAP pour l'immunogénicité (les 200 premiers enfants dans chaque centre). Les titres moyens géométriques (TMGs) d’anticorps anti-CS ont été quantifiés avant la vaccination, 1 mois après la 3^ème^ dose, immédiatement avant et 1 mois après la 4ème dose, aux mois d'étude 32 et 44, et à la fin de l'étude. Les TMGs d’anticorps anti-CS les plus élevés ont été mesurés 1 mois après la 3^ème^ dose (avec 99,9% d'enfants séropositifs). Au 20^ème^ mois (avant la 4^ème^ dose), les TMGs d’anticorps anti-CS avaient diminué de 18 fois chez les enfants qui avaient reçu RTS,S/AS01. Dans le groupe témoin, les TMGs d’anticorps anti-CS sont restés très faibles tout au long de l'étude, ce qui suggère qu'après une piqûre de moustique infecté, l'exposition aux sporozoïtes circulants avant qu'ils ne pénètrent dans les hépatocytes est trop faible pour permettre l'induction d'une réponse élevée en termes d’anticorps anti-CS (Figure 5). Dans la Figure 5, « RTS,S/AS01 » comprend les groupes RTS,S/AS01 3-dose et 4-dose avant l’administration de la 4^ème^ dose; « RTS,S/AS01 3-dose » correspond au groupe des enfants ayant reçu 3 doses de RTS,S/AS01 aux mois 0, 1 et 2 et le vaccin témoin au mois 20; « RTS,S/AS01 4-dose » correspond au groupe des enfants ayant reçu les 4 doses de RTS,S/AS01 aux mois 0, 1, 2 et 20; et « Témoin » correspond au groupe des enfants ayant reçu le vaccin témoin aux mois 0, 1 et 2 (pour les 3 premières doses) et au mois 20 (pour la 4^ème^ dose). « TMG » correspond au titre moyen géométrique, « CS » à la protéine circumsporozoite et « EU » aux unités ELISA. Les barres d'erreur indiquent les intervalles de confiance à 95%. La ligne verticale pointillée indique le moment de l’administration de la 4^ème^dose. Une régression linéaire effectuée après 18 mois de suivi (c-à-d. avant la 4^ème^ dose) a montré que les réponses anti-CS étaient significativement plus élevées chez les enfants qui étaient plus jeunes au moment de la vaccination (5–11 mois versus 12–17 mois) et chez les enfants qui vivaient dans un contexte de transmission plus forte [27]. Après l'administration de la 4^ème^ dose, les TMGs d’anticorps anti-CS ont augmenté de 9 fois, mais sans atteindre le niveau observé 1 mois après la 3^ème^ dose (Figure 5). Les plus faibles niveaux d'anticorps anti-CS observés après la 4^ème^ dose par rapport à la 3^ème^ dose pourraient soulever des questions par rapport à une diminution potentielle de la réponse immunitaire contre l’antigène CS lors de l’administration de doses ultérieures de RTS,S/AS01. Il convient toutefois de noter que la 4^ème^ dose améliore l’EV par rapport aux enfants n’ayant reçu que 3 doses de RTS,S/AS01. Le mécanisme responsable de cette immunogénicité réduite contre l’antigène CS après la 4^ème^ dose de RTS,S/AS01 est probablement différent du mécanisme observé pour les vaccins polysaccharidiques non-conjugués compte tenu des caractéristiques de la réponse immunitaire anti-CS, principalement l'induction de réponses des lymphocytes T CD4+ [14,19,50] et des lymphocytes B mémoire spécifiques au CS [50]. De plus, des cinétiques d’anticorps anti-CS semblables ont été observées précédemment dans une étude de phase II lorsque la 3^ème^ dose était retardée et RTS,S/AS01 était administré selon un schéma de vaccination à 0, 1 et 7 mois [17]. Des données récentes de cette étude suggèrent toutefois que les augmentations de concentration et d’avidité des anticorps observées après des vaccinations successives pourraient être associées à une réduction du risque de paludisme [51]. Bien qu’aucun seuil de protection n'ait pu être établi pour les anticorps anti-CS [19,20,52], les études de phase II chez les adultes [53] et les enfants de 5 à 17 mois [21] ont montré que les anticorps anti-CS et les lymphocytes T CD4+ spécifiques au CS sont associés à une protection contre les infections par *P. falciparum* et les cas de paludisme clinique. Dans une étude récente modélisant la relation entre la dynamique des titres d'anticorps anti-CS et la durée de l’EV en utilisant les données de l'essai de phase III, les titres d'anticorps anti-CS étaient prédictifs de l’EV contre les infections par *P. falciparum* à travers différents groupes d'âge et différents contextes de transmission du paludisme [54]. La dynamique des titres d'anticorps anti-CS après la vaccination suggère que la réponse humorale comporte une composante de courte durée et une composante de longue durée, avec une réponse de longue durée plus importante et une augmentation de la persistance des anticorps après une 4^ème^ vaccination. Ce modèle, associé à l'immunogénicité et aux données d’EV chez les enfants de 5 à 17 mois, renforce l'hypothèse selon laquelle l'administration de la 4^ème^ dose présente des avantages durables, même si les taux d'anticorps restent inférieurs à ceux observés 1 mois après la 3^ème^ dose.

### Innocuité et tolérabilité du vaccin

Dans chaque essai clinique, l'innocuité et la tolérabilité ont été évaluées pour la cohorte totale d’enfants vaccinés, comprenant tous les enfants ayant reçu au moins 1 dose de vaccin.

#### Réactogénicité

Dans la grande étude d'efficacité et d’innocuité de phase III, la réactogénicité chez les enfants de 5 à 17 mois a été évaluée chez les 200 premiers enfants recrutés dans chaque centre d'étude. L’incidence des symptômes locaux sollicités activement était légèrement plus élevée chez les enfants qui avaient reçu RTS,S/AS01 que dans le groupe témoin, et la douleur au point d'injection était le symptôme local le plus communément signalé (Tableau 2). Seuls quelques enfants ont présenté des symptômes locaux de grade 3 après avoir reçu RTS,S/AS01, et aucun n'a été signalé après une dose de vaccin témoin. L’incidence des symptômes généraux sollicités activement était aussi plus faible dans le groupe témoin (Tableau 2). Le symptôme général le plus souvent signalé dans les deux groupes était la fièvre (température axillaire = 37,5°C). Au cours des 3 premières doses, la fièvre a été signalée après 31,1% des doses de RTS,S/AS01 et 13,4% des doses de vaccin antirabique. Les symptômes de grade 3 étaient rares, mais une fièvre de grade 3 (température axillaire > 39,0°C) a été signalée après 2,5% des doses de RTS,S/AS01 [29]. Il n'y a pas eu d'augmentation notable de l'incidence des symptômes généraux sollicités activement après la 4^ème^ dose de RTS,S/AS01 par rapport aux 3 premières doses. La fièvre est demeurée le symptôme le plus fréquemment signalé à la fois après la 4^ème^ dose de RTS,S/AS01 et après la dose du vaccin témoin MenC au 20^ème^ mois (après respectivement 36,3% et 7,1% des doses). Après la 4^ème^ dose de RTS,S/AS01, de la fièvre de grade 3 a été signalée chez 5,3% des enfants [25].

**Tableau 2:** Incidence [% (intervalle de confiance à 95%)] des symptômes locaux et généraux sollicités activement pendant la période de suivi de 7 jours après les 3 premières doses (par dose) et après la 4^ème^ dose chez les enfants âgés de 5 à 17 mois au moment de la première vaccination (cohorte totale vaccinée pour la réactogénicité)

	Après les 3 premières doses		Après la 4^ème^ dose	
	RTS,S/AS01	Témoin	RTS,S/AS01	Témoin
Nombre de doses	4321	2128	641	633
**Symptômes locaux (au point d’injection)**				
Douleur				
Total	12,4 (11,4–13,4)	5,8 (4,9–6,9)	17,0 (14,2–20,1)	6,5 (4,7–8,7)
Grade 3	0,1 (0,0–0,2)	0,0 (0,0–0,2)	0,0 (0,0–0,6)	0,0 (0,0–0,6)
Rougeur				
Total	3,1 (2,6–3,7)	2,7 (2,0–3,5)	2,3 (1,3–3,8)	1,3 (0,5–2,5)
Grade 3	0,2 (0,1–0,3)	0,0 (0,0–0,2)	0,5 (0,1–1,4)	0,0 (0,0–0,6)
Gonflement				
Total	9,6 (8,7–10,5)	7,6 (6,5–8,8)	6,6 (4,8–8,8)	4,7 (3,2–6,7)
Grade 3	0,7 (0,5–1,0)	0,0 (0,0–0,2)	1,4 (0,6–2,6)	0,0 (0,0–0,6)
**Symptômes généraux**				
Somnolence				
Total	6,6 (5,9–7,4)	4,4 (3,5–5,3)	8,6 (6,5–11,0)	3,3 (2,1–5,0)
Grade 3	0,1 (0,0–0,3)	0,0 (0,0–0,2)	0,2 (0,0–0,9)	0,0 (0,0–0,6)
**Irritabilité**				
Total	11,5 (10,5–12,4)	5,3 (4,4–6,3)	9,8 (7,6–12,4)	2,8 (1,7–4,5)
Grade 3	0,1 (0,0–0,2)	0,0 (0,0–0,2)	0,2 (0,0–0,9)	0,0 (0,0–0,6)
Perte d’appétit				
Total	11,4 (10,4–12,3)	7,4 (6,3–8,6)	10,3 (8,1–12,9)	3,3 (2,1–5,0)
Grade 3	0,1 (0,0–0,3)	0,0 (0,0–0,2)	0,2 (0,0–0,9)	0,0 (0,0–0,6)
**Température (axillaire)**				
≥37,5 °C	31,1 (29,7–32,5)	13,4 (12,0–14,9)	36,3 (32,6–40,2)	7,1 (5,2–9,4)
>39,0 °C	2,5 (2,1–3,1)	1,1 (0,7–1,7)	5,3 (3,7–7,3)	0,8 (0,3–1,8)

RTS,S/AS01 (pour les 3 premières doses) : les enfants ayant reçu au moins une dose de RTS,S/AS01 ; RTS,S/AS01 4-dose : les enfants ayant reçu les 4 doses de RTS,S/AS01 aux mois 0, 1, 2 et 20 ; Témoin : les enfants ayant reçu le vaccin témoin antirabique aux mois 0, 1 et 2 (pour les 3 premières doses) et MenC au mois 20 (pour la 4^ème^ dose)

#### Evènements indésirables et signaux relatifs à la sécurité du vaccin

Aucune différence significative en termes d'incidence globale d’évènements indésirables (EIs) n'a été observée entre les groupes RTS,S/AS01 et témoin chez les enfants de 5 à 17 mois. Au moins 1 EI a été signalée spontanément dans les 30 jours après une des 3 premières doses chez 86,1% (IC à 95%: 84,2 – 87,8) des enfants ayant reçu le vaccin RTS,S/AS01 et chez 86,8% (IC à 95% : 84,1 – 89,2) des enfants du groupe témoin [29]. La proportion d’enfants signalant spontanément des EIs dans les 30 jours après la 4^ème^ dose était de 36,2% (IC à 95% : 32,5 – 40,0) dans le groupe RTS,S/AS01 4 doses, 32,1% (IC à 95%: 28,5–35,9) dans le groupe RTS,S/AS01 3 doses, et 34,0% (IC à 95%: 30,3 – 37,8) dans le groupe témoin. Les infections des voies respiratoires supérieures, le paludisme et la fièvre étaient les EIs les plus fréquemment signalées spontanément dans les 3 groupes pendant toute la durée de l'étude [25]. En plus des symptômes locaux activement sollicités et considérés comme étant liés à la vaccination, une évaluation des EIs signalées spontanément dans les 30 jours suivant la vaccination a identifié 3 EIs potentiellement liées à la vaccination avec RTS,S/AS01: l’induration au point d'injection, les vomissements et la diarrhée [10]. L'incidence des évènements indésirables graves (EIGs) sur la durée de l'étude est présentée dans le Tableau 3. L'incidence globale des EIGs dans l'essai de phase III était légèrement plus faible chez les enfants ayant reçu RTS,S/AS01 que dans le groupe témoin, conformément aux résultats de l'analyse combinée des données d’innocuité issues des essais de phase II [55] dans lesquelles les vaccins RTS,S/AS01 et RTS,S/AS02 étaient significativement associés à une diminution du risque relatif de EIG par rapport au groupe témoin. Une diminution de l'incidence des pneumonies signalées comme EIGs a également été notée dans l'analyse des étude de phase II [55], mais cette observation n'a pas été confirmée dans l'essai de phase III, où la pneumonie a été évaluée en tant que critère d'efficacité. Comme on pouvait s'y attendre pour une population pédiatrique africaine, le paludisme, la pneumonie, la gastro-entérite et l'anémie étaient des EIGs fréquemment signalées [25]. Les cas de pneumonie et de gastro-entérite étaient répartis de manière homogène entre les groupes, alors que l'incidence du paludisme et de l'anémie était plus faible dans les groupes RTS,S/AS01. Toutes les convulsions apparaissant dans les 30 jours suivant la vaccination ont été reportées comme des EIGs afin de maximiser la collecte de données concernant les crises convulsives généralisées qui avaient été identifiées précédemment comme des EIs présentant un intérêt particulier [55]. Bien que ces observations n'étaient pas statistiquement significatives, un taux plus élevé de convulsions (niveaux 1-3 selon la définition du groupe de travail collaboratif de Brighton) dans les 7 jours suivant les 3 premières doses de vaccin a été observé dans le groupe RTS,S/AS01 (1,04 pour 1 000 doses) par rapport au groupe témoin ayant reçu le vaccin antirabique (0,57 pour 1 000 doses), avec un risque relatif (RR) de 1,8 (IC à 95%: 0,6 – 4,9). Endéans les 30 jours après la vaccination, le taux global d'enfants ayant présenté des convulsions fébriles était similaire dans les groupes RTS,S/AS01 (39 enfants sur 5 948; 1,0%) et témoin (17 enfants sur 2 974; 0,8%) [6]. L'incidence des convulsions dans les 7 jours après la 4ème dose était de 2,5 pour 1 000 doses de RTS,S/AS01 et de 0,4 pour 1 000 doses dans le groupe témoin [6]. Toutes les convulsions fébriles survenues dans les 7 jours suivant la vaccination ont été considérées comme potentiellement liées à la vaccination en se basant sur la relation temporelle et une explication physiologique plausible. Dans l'essai d'efficacité de phase III, un déséquilibre numérique des cas de méningite a été observé après les 3 premières doses. Dans les 20 mois de suivi après la 1^ère^ dose, 16 cas ont été identifiés chez les 5 948 enfants ayant reçu RTS,S/AS01, et 1 cas chez les 2 974 enfants du groupe témoin (RR = 8,0 (IC à 95%: 1,1 – 60,3)) [6,27]. Du 21^ème^ mois jusqu’à la fin de l’étude, 5 cas supplémentaires de méningite ont été signalés dans les groupes RTS,S/AS01 (3 dans le groupe 3 doses et 2 dans le groupe 4 doses) et aucun dans le groupe témoin [25,36]. Pendant toute la période de suivi d'environ 4 ans, 11 cas ont été signalés dans le groupe RTS,S/AS01 4 doses, 10 cas dans le groupe RTS,S/AS01 3 doses, et 1 cas dans le groupe témoin [25].

**Tableau 3:** Pourcentage [% (intervalle de confiance à 95%)] de participants avec des évènements indésirables graves signalés pendant une période de suivi de 18 mois après la 3^ème^ dose et jusqu'à la fin de l'étude de phase III chez les enfants âgés de 5 à 17 mois (cohorte totale vaccinée)

	Pendant un suivi de 18 mois		Pendant toute la durée de l'étude		
	RTS,S/AS01	Témoin	RTS,S/AS01-3 dose	RTS,S/AS01-4 dose	Témoin
Nombre de doses	5949	2974	2972	2976	2974
Au moins une EIG	18,6 (17,6–19,6)	22,7 (21,2–24,3)	25,3 (23,7–26,9)	24,2 (22,7–25,8)	28,4 (26,8–30,1)
Au moins une EIG autre que le paludisme	17,6 (16,6–18,6)	21,2 (19,7–22,7)	23,7 (22,2–25,3)	22,6 (21,1-24,2)	26,4 (24,8–28,0)
Décès	1,2 (1,0–1,6)	1,1 (0,8–1,6)	1,7 (1,3–2,3)	2,0 (1,6–2,6)	1,5 (1,1–2,1)
Au moins une EIG liée au vaccin	0,2 (0,1–0,3)	0,0 (0,0–0,2)	0,1 (0,0–0,3)	0,3 (0,1–0,5)	0,0 (0,0–0,2)
Convulsion fébrile	3,8 (3.3–4.3)	3,8 (3,1–4,5)	6,2 (5,4–7,1)	5,3 (4,6–6,2)	5,5 (4,7–6,4)

EIG, évènement indésirable grave; RTS,S/AS01 (pour les 3 premières doses): les enfants ayant reçu au moins une dose de RTS,S/AS01; RTS,S/AS01 3-dose, les enfants ayant reçu 3 doses de RTS,S/AS01 aux mois 0, 1 et 2 et le vaccin témoin Men C au mois 20; RTS,S/AS01 4-dose: les enfants ayant reçu les 4 doses de RTS,S/AS01 aux mois 0, 1, 2 et 20; Témoin: les enfants ayant reçu le vaccin témoin antirabique aux mois 0, 1 et 2 (pour les 3 premières doses) et Men C au mois 20 (pour la 4ème dose).

Par contre, aucune disparité n'a été observée chez les enfants âgés de 6 à 12 semaines. De plus, aucune relation temporelle avec la vaccination n'a été établie et les étiologies des méningites étaient diverses. Une grande partie des cas (11) ont été identifiés dans le centre d’étude de Lilongwe au Malawi, mais aucune épidémie de méningite n'a été signalée dans ce centre. Ce déséquilibre est très probablement le fruit du hasard et peut être expliqué par le faible nombre de cas dans le groupe témoin (1 seul cas chez 2 974 enfants suivi durant 4 ans en moyenne, comparé à 6 cas chez les 2 179 nourrissons du groupe témoin suivi durant 3 ans en moyenne). Néanmoins, la méningite est considérée comme un risque potentiel qui sera étroitement surveillé et évalué dans les essais de phase IV. Dans des analyses supplémentaires réalisées pour évaluer l'effet rebond potentiel du vaccin RTS,S/AS01, l'incidence des hospitalisations dues au paludisme grave avec > 5 000 parasites par mm^3^ et coma (un Score de Coma de Blantyre (SCB) < 3), compatible avec le paludisme cérébral, semblait plus élevée dans les groupes RTS,S/AS01 que dans le groupe témoin. Comme cette analyse n'était pas planifiée dans le protocole, le diagnostic de paludisme cérébral n'a pas été confirmé cliniquement et le faible SCB pourrait avoir été la conséquence d'autres maladies concomitantes. Au cours des 20 premiers mois, avant la 4^ème^ dose, 22 cas d’hospitalisations dues au paludisme avec un faible SCB ont été identifiés parmi les 5 948 enfants ayant reçu RTS,S/AS01, tandis que 6 cas sont apparus parmi les 2 974 enfants du groupe témoin. Du 21^ème^ mois jusqu'à la fin de l'étude, 9 autres cas ont été identifiés parmi les 2 719 enfants ayant reçu 3 doses de RTS,S/AS01, 12 cas parmi les 2 681 enfants ayant reçu 4 doses de RTS,S/AS01 et 4 cas parmi les 2 702 enfants du groupe témoin [36]. Une telle disparité n'a pas été retrouvée chez les enfants âgés de 6 à 12 semaines. Il est difficile de trouver une explication biologiquement plausible pour un effet direct d’un vaccin pré-érythrocytaire sur la pathogenèse du paludisme grave survenant pendant le stade sanguin. En effet, le délai entre la vaccination et l’apparition des cas ne supporte ni un effet direct de la vaccination, ni un effet indirect lié à l'acquisition tardive de l'immunité naturelle. L'augmentation de l’incidence du paludisme grave mentionnée précédemment dans le groupe 3 doses était principalement due à un nombre excessif de cas avec d’autres marqueurs de sévérité qu’un SCB faible. La disparité observée peut donc être une découverte fortuite, mais des changements dans la répartition des âges et la présentation clinique du paludisme grave due à la protection conférée initialement par le vaccin et l'acquisition retardée de l'immunité naturelle ne peuvent pas être exclu. L'incidence du paludisme grave (y compris le paludisme cérébral) sera étroitement surveillée dans les études de phase IV. En raison de la qualité des soins médicaux fournis aux participants, la mortalité observée dans l’étude n'est pas représentative de la mortalité globale en situation réelle en Afrique et les résultats des analyses de mortalité doivent être interprétés avec prudence. À la demande de l'OMS, la mortalité toutes causes confondues a néanmoins été évaluée par sexe dans une analyse *post-hoc*, et le taux de mortalité semblait être 2 fois plus élevé chez les filles ayant reçu RTS,S/AS01 que chez les filles ayant reçu le vaccin témoin (123/5 091 vs 33/2 603, tous âges confondus). Ce déséquilibre était largement dû à la faible mortalité féminine dans le groupe témoin [56,57]. Une telle disparité n'a pas été observée chez les garçons. De plus, les taux de mortalité en fonction du sexe ne sont pas disponibles pour les pays/communautés dans lesquels l'étude a été réalisée. Cette analyse, qui n'était pas prévue par le protocole, a pu être affectée par des facteurs confondants, tels que l’administration concomitante des vaccins du PEV, la malnutrition et le statut VIH. Aucun des décès n'a été considéré comme lié à la vaccination par les investigateurs de l'étude et la différence n'était pas liée à un événement spécifique mais à des causes multiples, comprenant des maladies infectieuses, des traumatismes et le paludisme. L’implémentation pilote fournira des informations supplémentaires sur l'impact de RTS,S/AS01 sur les disparités liées au sexe et les taux de mortalité globaux.

## Conclusion

Nous avons montré qu’un schéma vaccinal à 4 doses du vaccin RTS,S/AS01 est efficace et offre le plus de bénéfices contre le paludisme clinique et le paludisme grave chez les enfants âgés de 5 à 17 mois au moment de la première vaccination. Toutes les prévisions de modélisation suggèrent qu'un nombre substantiel de cas de paludisme clinique, de paludisme grave et de décès dus au paludisme pourrait être évité en administrant RTS,S/AS01 à 6, 7½, 9 et 27 mois, en particulier dans les situations de transmission modérée à forte en Afrique subsaharienne. Nous avons également montré que le vaccin a un profil d’innocuité acceptable dans la catégorie d'âge visée pour l’implémentation. Les EIs identifiées comme potentiellement liées à la vaccination au cours des essais cliniques ont également été rapportées après l'administration d'autres vaccins homologués. Le seul risque identifié est l'apparition de convulsions fébriles dans les 7 jours suivant la vaccination chez les enfants âgés de 5 mois ou plus. Des incertitudes concernant certaines observations, telles que les méningites, le paludisme cérébral et la mortalité par sexe, seront évaluées en profondeur dans les études de phase IV et pendant l’implémentation pilote.

### Etat des connaissances actuelles sur le sujet

- Malgré un déclin récent de l’incidence du paludisme en Afrique, cette maladie reste endémique dans beaucoup de régions en Afrique et a causé, selon les estimations, 438.000 décès en 2015, principalement parmi les enfants âgés de moins de 5 ans;
- Les succès réalisés dans les régions de haute transmission en Afrique Sub-Saharienne sont menacés par un accès limité aux soins médicaux, spécialement dans les zones rurales aux infrastructures précaires, la propagation de souches de *Plasmodium* résistantes à l’artémisinine à partir de l’Asie du Sud-Est et la propagation des moustiques résistants aux insecticides;
- Actuellement, les programmes de contrôle du paludisme combinent plusieurs interventions partiellement efficaces pour maximiser leurs impacts; même avec une efficacité partielle, un vaccin contre le paludisme peut mener à un impact substantiel sur la santé publique.

### Contribution de notre étude à la connaissance

- L’essai clinique a montré qu’un schéma à 4 doses du vaccin RTS,S/AS01 est efficace et procure le bénéfice le plus important chez les enfants âgés de 5 à 17 mois à la première dose vivants dans les régions de moyenne à haute endémicité;
- Des prévisions de modélisation estiment que, sur 15 ans, en moyenne 1 décès et 230 cas de paludisme peuvent être évités pour 200 enfants vaccinés avec 4 doses de RTS,S/AS01 si le vaccin est implémenté dans des régions avec une prévalence de parasitémie de *P. falciparum* de 10% ou plus;
- L’implémentation pilote et les études de phase 4 permettront d’évaluer l’innocuité du vaccin, son impact sur la mortalité et la faisabilité opérationnelle d’un schéma à 4 doses dans le cadre d’une vaccination de routine à large échelle en Afrique Sub-Saharienne.

## Conflits d’intérêts

Les auteurs ne déclarent aucun conflit d'intérêts.

## Annexe

**Annexe 1**: encadré: principales définitions des cas de paludisme cliniques et graves dans l’étude de Phase III

## References

[1] WHO (2015). World Malaria Report.

[2] Noor AM, Kinyoki DK, Mundia CW (2014). The changing risk of Plasmodium falciparum malaria infection in Africa: 2000-10: a spatial and temporal analysis of transmission intensity. Lancet.

[3] Bhatt S, Weiss DJ, Cameron E (2015). The effect of malaria control on Plasmodium falciparum in Africa between 2000 and 2015. Nature.

[4] Pluess B, Tanser FC, Lengeler C, Sharp BL (2010). Indoor residual spraying for preventing malaria. Cochrane Database Syst Rev.

[5] Protopopoff N, Matowo J, Malima R (2013). High level of resistance in the mosquito Anopheles gambiae to pyrethroid insecticides and reduced susceptibility to bendiocarb in north-western Tanzania. Malar J.

[6] European Medicine Agencies, CHMP (2015). Assessment report Mosquirix.

[7] WHO (2016). Malaria vaccine: WHO position paper-January 2016. Wkly Epidemiol Rec.

[8] Cohen J, Nussenzweig V, Nussenzweig R (2010). From the circumsporozoite protein to the RTS, S/AS candidate vaccine. Hum Vaccin.

[9] Regules JA, Cummings JF, Ockenhouse CF (2011). The RTS,S vaccine candidate for malaria. Expert Rev Vaccines.

[10] (2015). Mosquirix Product Information.

[11] Birkett AJ (2016). Status of vaccine research and development of vaccines for malaria. Vaccine.

[12] WHO (2014). WHO Expert Committee on Biological Standardization 63rd report, Annex 3: Guidelines on the quality, safety and efficacy of recombinant malaria vaccines targeting the pre-erythrocytic and blood stages of Plasmodium falciparum.

[13] Agnandji ST, Fernandes JF, Bache EB, Ramharter M (2015). Clinical development of RTS,S/AS malaria vaccine: a systematic review of clinical Phase I-III trials. Future Microbiol.

[14] Ansong D, Asante KP, Vekemans J (2011). T cell responses to the RTS,S/AS01(E) and RTS,S/AS02(D) malaria candidate vaccines administered according to different schedules to Ghanaian children. PLoS One.

[15] Owusu-Agyei S, Ansong D, Asante K (2009). Randomized controlled trial of RTS,S/AS02D and RTS,S/AS01E malaria candidate vaccines given according to different schedules in Ghanaian children. PLoS One.

[16] Olotu A, Lusingu J, Leach A (2011). Efficacy of RTS,S/AS01E malaria vaccine and exploratory analysis on anti-circumsporozoite antibody titres and protection in children aged 5-17 months in Kenya and Tanzania: a randomised controlled trial. Lancet Infect Dis.

[17] Agnandji ST, Asante KP, Lyimo J (2010). Evaluation of the safety and immunogenicity of the RTS,S/AS01E malaria candidate vaccine when integrated in the expanded program of immunization. J Infect Dis.

[18] Bejon P, Lusingu J, Olotu A (2008). Efficacy of RTS,S/AS01E vaccine against malaria in children 5 to 17 months of age. N Engl J Med.

[19] Ndungu FM, Mwacharo J, Kimani D (2012). A statistical interaction between circumsporozoite protein-specific T cell and antibody responses and risk of clinical malaria episodes following vaccination with RTS,S/AS01E. PLoS One.

[20] Bejon P, Cook J, Bergmann-Leitner E (2011). Effect of the pre-erythrocytic candidate malaria vaccine RTS,S/AS01E on blood stage immunity in young children. J Infect Dis.

[21] Olotu A, Moris P, Mwacharo J (2011). Circumsporozoite-specific T cell responses in children vaccinated with RTS,S/AS01E and protection against P falciparum clinical malaria. PLoS One.

[22] Lusingu J, Olotu A, Leach A (2010). Safety of the Malaria Vaccine Candidate, RTS,S/AS01E in 5 to 17 Month Old Kenyan and Tanzanian Children. PLoS One.

[23] Asante KP, Abdulla S, Agnandji S (2011). Safety and efficacy of the RTS,S/AS01E candidate malaria vaccine given with expanded-programme-on-immunisation vaccines: 19 month follow-up of a randomised, open-label, phase 2 trial. Lancet Infect Dis.

[24] Leach A, Vekemans J, Lievens M (2011). Design of a phase III multicenter trial to evaluate the efficacy of the RTS,S/AS01 malaria vaccine in children across diverse transmission settings in Africa. Malar J.

[25] RTS,S Clinical Trials Partnership (2015). Efficacy and safety of RTS,S/AS01 malaria vaccine with or without a booster dose in infants and children in Africa: final results of a phase 3, individually randomised, controlled trial. Lancet.

[26] Lievens M, Aponte JJ, Williamson J (2011). Statistical methodology for the evaluation of vaccine efficacy in a phase III multi-centre trial of the RTS, S/AS01 malaria vaccine in African children. Malar J.

[27] RTS,S Clinical Trials Partnership (2014). Efficacy and safety of the RTS,S/AS01 malaria vaccine during 18 months after vaccination: a phase 3 randomized, controlled trial in children and young infants at 11 African sites. PLoS Med.

[28] Neafsey DE, Juraska M, Bedford T (2015). Genetic diversity and protective efficacy of the RTS, S/AS01 malaria vaccine. N Engl J Med.

[29] RTS,S Clinical Trials Partnership (2011). First results of phase 3 trial of RTS,S/AS01 malaria vaccine in African children. N Engl J Med.

[30] Swysen C, Vekemans J, Bruls M (2011). Development of standardized laboratory methods and quality processes for a phase III study of the RTS, S/AS01 candidate malaria vaccine. Malar J.

[31] Vekemans J, Marsh K, Greenwood B (2011). Assessment of severe malaria in a multicenter, phase III, RTS, S/AS01 malaria candidate vaccine trial: case definition, standardization of data collection and patient care. Malar J.

[32] RTS,S Clinical Trials Partnership (2012). A phase 3 trial of RTS,S/AS01 malaria vaccine in African infants. N Engl J Med.

[33] Otieno L, Oneko M, Otieno W (2016). Safety and immunogenicity of RTS,S/AS01 malaria vaccine in infants and children with WHO stage 1 or 2 HIV disease: a randomised, double-blind, controlled trial. Lancet Infect Dis.

[34] Umeh R, Oguche S, Oguonu T (2014). Immunogenicity and safety of the candidate RTS,S/AS01 vaccine in young Nigerian children: a randomized, double-blind, lot-to-lot consistency trial. Vaccine.

[35] Vandoolaeghe P, Schuerman L (2016). The RTS, S/AS01 malaria vaccine in children 5 to 17 months of age at first vaccination. Expert Rev Vaccines.

[36] Joint Technical Expert Group & World Health Organization (2015). Background paper on the RTS,S/AS01 malaria vaccine.

[37] Olotu A, Fegan G, Wambua J (2013). Four-year efficacy of RTS,S/AS01E and its interaction with malaria exposure. N Engl J Med.

[38] Olotu A, Fegan G, Wambua J (2016). Seven-year efficacy of RTS,S/AS01 malaria vaccine among young African children. N Engl J Med.

[39] Bejon P, White MT, Olotu A (2013). Efficacy of RTS,S malaria vaccines: individual-participant pooled analysis of phase 2 data. Lancet Infect Dis.

[40] Reyburn H, Mbatia R, Drakeley C (2005). Association of transmission intensity and age with clinical manifestations and case fatality of severe Plasmodium falciparum malaria. JAMA.

[41] Taylor T, Olola C, Valim C (2006). Standardized data collection for multi-center clinical studies of severe malaria in African children: establishing the SMAC network. Trans R Soc Trop Med Hyg.

[42] Jallow M, Casals-Pascual C, Ackerman H (2012). Clinical features of severe malaria associated with death: a 13-year observational study in the Gambia. PLoS One.

[43] Dondorp AM, Fanello CI, Hendriksen IC (2010). Artesunate versus quinine in the treatment of severe falciparum malaria in African children (AQUAMAT): an open-label, randomised trial. Lancet.

[44] Hamel MJ, Oneko M, Williamson J A marked reduction in mortality among participants in a clinical trial that removed barriers to care and implemented national case management guidelines.

[45] Alloueche A, Milligan P, Conway DJ (2003). Protective efficacy of the RTS, S/AS02 Plasmodium falciparum malaria vaccine is not strain specific. The American journal of tropical medicine and hygiene.

[46] Enosse S, Dobaño C, Quelhas D (2006). RTS, S/AS02A malaria vaccine does not induce parasite CSP T cell epitope selection and reduces multiplicity of infection. PLOS Clin Trial.

[47] Waitumbi JN, Anyona SB, Hunja CW (2009). Impact of RTS,S/AS02(A) and RTS,S/AS01(B) on genotypes of P falciparum in adults participating in a malaria vaccine clinical trial. PLoS One.

[48] Penny MA, Verity R, Bever CA (2016). Public health impact and cost-effectiveness of the RTS,S/AS01 malaria vaccine: a systematic comparison of predictions from four mathematical models. Lancet.

[49] Penny MA, Pemberton-Ross P, Smith TA (2015). The time-course of protection of the RTS,S vaccine against malaria infections and clinical disease. Malar J.

[50] Agnandji ST, Fendel R, Mestre M (2011). Induction of Plasmodium falciparum-specific CD4+ T cells and memory B cells in Gabonese children vaccinated with RTS,S/AS01(E) and RTS,S/AS02(D). PLoS One.

[51] Ajua A, Lell B, Agnandji ST (2015). The effect of immunization schedule with the malaria vaccine candidate RTS,S/AS01E on protective efficacy and anti-circumsporozoite protein antibody avidity in African infants. Malar J.

[52] Horowitz A, Hafalla JC, King E (2012). Antigen-specific IL-2 secretion correlates with NK cell responses after immunization of Tanzanian children with the RTS,S/AS01 malaria vaccine. J Immunol.

[53] Kester KE, Cummings JF, Ofori-Anyinam O (2009). Randomized, double-blind, phase 2a trial of falciparum malaria vaccines RTS,S/AS01B and RTS,S/AS02A in malaria-naive adults: safety, efficacy, and immunologic associates of protection. J Infect Dis.

[54] White MT, Verity R, Griffin JT (2015). Immunogenicity of the RTS,S/AS01 malaria vaccine and implications for duration of vaccine efficacy: secondary analysis of data from a phase 3 randomised controlled trial. Lancet Infect Dis.

[55] Vekemans J, Guerra Y, Lievens M (2011). Pooled analysis of safety data from pediatric Phase II RTS,S/AS malaria candidate vaccine trials. Hum Vaccin.

[56] Guerra Mendoza Y, Garric E, Schuerman L (2016). Discussion of meningitis, cerebral malaria and all-cause mortality followind immunization of children with the RTS,S/AS01 malaria vaccine in the phase III efficacy trial.

[57] Klein SL, Shann F, Moss WJ (2016). RTS,S Malaria Vaccine and Increased Mortality in Girls. MBio.

